# Assessment and Distribution of Runs of Homozygosity in Horse Breeds Representing Different Utility Types

**DOI:** 10.3390/ani12233293

**Published:** 2022-11-25

**Authors:** Tomasz Szmatoła, Artur Gurgul, Igor Jasielczuk, Ewa Oclon, Katarzyna Ropka-Molik, Monika Stefaniuk-Szmukier, Grazyna Polak, Iwona Tomczyk-Wrona, Monika Bugno-Poniewierska

**Affiliations:** 1 Center for Experimental and Innovative Medicine, University of Agriculture in Krakow, Rędzina 1c, 30-248 Kraków, Poland; 2 Department of Animal Molecular Biology, National Research Institute of Animal Production, Krakowska 1, 32-083 Balice, Poland; 3 Office of the Director for Scientific Affairs, National Research Institute of Animal Production, Krakowska 1, 32-083 Balice, Poland; 4 Department of Horse Breeding, National Research Institute of Animal Production, Krakowska 1, 32-083 Balice, Poland; 5 Department of Animal Reproduction, Anatomy and Genomics, University of Agriculture in Kraków, al. Mickiewicza 24/28, 30-059 Kraków, Poland

**Keywords:** ROH, microarray, inbreeding, autozygosity

## Abstract

**Simple Summary:**

In recent years, the concept of runs of homozygosity (ROH) has become increasingly important in the genetic studies of various animal species, due to its advantages in estimating inbreeding and identifying regions under selection pressure. In this study, ROH distribution in six horse breeds belonging to three horse types (primitive, light, and draft) was examined. The presented results showed diverse differences in the length, number, and frequency of ROH between the analyzed breeds. Moreover, genomic inbreeding coefficients (F_ROH_) showed a higher level of inbreeding in light and primitive horses in comparison to draft horses. Regarding ROH islands, which are regions of the genome characterized by a high frequency of ROH and thus may represent signals of selection events, we observed several genes with confirmed effects on major horse breed characteristics. In addition, ROH regions of zero frequency were analyzed and these regions also spanned across genes involved in important breed characteristics. The presented results, especially regarding ROH islands and no-ROH regions, can be used as a basis for further research concerning the identification of genes and markers that determine important horse breed characteristics.

**Abstract:**

The present study reports runs of homozygosity (ROH) distribution in the genomes of six horse breeds (571 horses in total) representing three horse types (primitive, light, and draft horses) based on the 65k Equine BeadChip assay. Of major interest was the length, quantity, and frequency of ROH characteristics, as well as differences between horse breeds and types. Noticeable differences in the number, length and distribution of ROH between breeds were observed, as well as in genomic inbreeding coefficients. We also identified regions of the genome characterized by high ROH coverage, known as ROH islands, which may be signals of recent selection events. Eight to fourteen ROH islands were identified per breed, which spanned multiple genes. Many were involved in important horse breed characteristics, including *WFIKNN2*, *CACNA1G*, *STXBP4*, *NOG*, *FAM184B*, *QDPR*, *LCORL*, and the zinc finger protein family. Regions of the genome with zero ROH occurrences were also of major interest in specific populations. Depending on the breed, we detected between 2 to 57 no-ROH regions and identified 27 genes in these regions that were common for five breeds. These genes were involved in, e.g., muscle contractility (*CACNA1A*) and muscle development (*miR-23*, *miR-24*, *miR-27*). To sum up, the obtained results can be furthered analyzed in the topic of identification of markers unique for specific horse breed characteristics.

## 1. Introduction

In recent years, a great interest arose in the subject of runs of homozygosity—especially in animal genomics. Runs of homozygosity (ROH) are vast homozygous regions in the genome, formed when both haplotypes inherited from the parents are identical. Moreover, these haplotypes tend to be of a common ancestral origin and thus can describe genetic relatedness among individuals and animal autozygosity [1]. Thanks to this, ROH analysis can be used as a tool to estimate inbreeding by assessing the genomic inbreeding coefficient (F_ROH_), calculated as the ratio of the sum of all ROH lengths per individual to the total length of the genome [2,3]. The advantages of using genomic inbreeding coefficients such as F_ROH_ in comparison to classical ways of its estimation (F_PED_) include more accurate prediction of inbreeding phenomena and the possibility to assess individual autozygosity levels without any pedigree information [1,3,4,5]. ROH can also be used for tracking the history of recent and ancient selection, by assessing the extent and frequency of ROH regions [6,7]. In general, long ROH describes recent inbreeding events while short ROH indicates the presence of more ancient consanguinity, which is often not included in the recording processes of pedigrees. The observation that ROH can create unique patterns across genomes of various populations, led to the utilization of a new term—‘ROH islands’ or ‘ROH hotspots’ [8,9]. ROH islands are commonly observed, can be specific to a population of interest, and are potentially associated with natural or artificial selection events [10]. Thus, ROH can be also used to describe the phenomenon of selective sweeps and to identify genomic regions that are a subject of selection pressure. Moreover, regions of the genome characterized by the absence of ROH in a respective population could also be of interest.

The first study concerning ROH was carried out over a decade ago in human populations, describing the frequency and distribution of ROH in the human genome [11]. Soon after, research on bovines was conducted by Solkner et al. [12] and Ferencakovic et al. [4]. Since then, interest in the ROH topic escalated in cattle genomics, which resulted in several studies describing various methods of identification and potential usage of ROH [1,5,13,14].

Selection across centuries of human demands and historical societal priorities created the present shape of modern horse breeds in less than 4000 years [15]. It is widely recognized that Arabians are one of the oldest and most highly influential breeds of horses in the world [16]. Their exact origin is not established, but there is some evidence that most of the present breeding stock comes from the northern Arabian Peninsula and the Syrian steppe, where they were traditionally bred by Bedouins. Since the 18th century, this breed has been widely distributed across Europe and currently several Arabian horse populations are distributed worldwide. These populations differ to a greater or lesser extent, e.g., the Polish, Russian, French, German and American populations, as well as populations of the Middle East such as Egyptian, Syrian, Tunisian, and Arab-Saudi [17]. The shape of the modern population would not be possible without consistent selection focused on traits associated with the desired type (head and neck conformation) alongside maintaining the proper and balanced physique of leading individuals to compete in various equestrian disciplines and racing. The nineteenth century also increased the popularity of breeding Thoroughbred (TB) horses—the first breed selected only for racing. Due to this trend, local mares were the basis of most saddle horse breeding in Europe with no exception of local polish mares [18]. Thus, similar breeding procedures were maintained in Poland. In the stages of creating the Małopolska breed, the native polish mares associated with the Malopolska region were crossed with TBs and Arabian stallions, as well as certain Hungarian breeds such as Nonius, Shagya and Gidran [19] to form a well-balanced riding horse [20]. As of now around 500 horses are maintained in the Polish genetic resources conservation program. In turn, horses used for heavy industrial purposes, road transportation, and in the military (Polish Heavy Draught) were refined with imported cold-blood breeds such as Ardennes, Belgian, German cold-blood, Percheron, Breton, Norwegian Fjord, and Slovenian. However, with the progress of mechanization, the population of heavy-working horse breeds became limited. Nowadays, these horse breeds are being maintained under the EU Common Agricultural Policy (CAP) and Animal Genetic Resources Conservation Programs to preserve the gene pool and to protect the horses’ biodiversity and cultural heritage [21]. In the case of primitive horses such as Polish Konik and Huculs, the breeds usually preserve their natural abilities to cope with low food requirements and their adaptation to difficult environmental conditions. Mostly, breeding strategies assume very little interference in the selection process—especially in reserve farms of Polish Koniks (Bialowieza stud farm). Additionally, the Carpathian Mountain origin of Hucul horses represents a different selection pressure than that of Polish Koniks [22,23].

The Polish Konik horse is a native horse breed belonging to a primitive type characterized by a valuable genetic reserve inherited from the Tarpans, thus presenting unique abilities of adaptation to the environment, good general health, feed efficiency and high fertility. Polish Konik horses are easily recognizable by their coat color, a Dun type with a predominant black Grullo color, as well as primitive body conformation. During World War II the population of Polish Konik horses was strongly diminished which resulted with a rebuilding of the population from a small number of remaining animals. As of today around 1800 animals are maintained in the Polish genetic resources conservation program [23,24]. A second primitive breed used in this study is Hucul which is described as horses remarkably adapted to mountainous difficult conditions and a great example of a natural breeding relict. These horses are known for their longevity, courage and high endurance. Moreover, they are one of the oldest primitive breeds originating from crossing with breeds such as Tarpans, Przewalski horses, Tatar and Turkish horses, as well as horses with Noric blood. As of today close to 1300 horses are maintained in the Polish genetic resources conservation program which is aimed to maintain their genetic variablity [23,25]. In regard to draft horses, two breeds Sokolski and Sztumski were used in this study, both of which are maintained within Polish genetic resource conservation program. Sokolski horses were bred mostly in the north-east regions of Poland which are considered to have a harsh climate and poorer quality of soils in regard to other parts of Poland. The formation of the breed was based on primitive local horses with an addition of imported Adrennes and Breton sires which led to development of a strong working horse characterized by a think fat cover, well-defined tendons, mild temperament and high endurance. Sztumski horses, which are the largest and heaviest cold-blooded horses maintained in Poland, were formed by crossbreeding of local horses with Adrennes and Belgian sires. They were mostly bred in the areas of Powisle, Warmia and Mazury which are categorized by difficult to cultivate soil. This led to a formation of a strong working horse breed, characterized by a larger caliber and even thicker fat cover compared to Sokolski horses. Regarding the number of horses maintained under Polish genetic resources conservation program: 1200 for Sokolski and 1300 for Sztumski horses. [23,26]. More information about the breeds used in this study can be found in: for Sokolski and Sztumski [21,23,26], for Polish Konik and Hucul [23,24,25] and for Malopolski horses [23,27].

Thus, this study aimed to assess the distribution of runs of homozygosity in the genomes of six horse breeds that represent three horse types: primitive (Polish Konik and Hucul horses), light (Arabian and Malopolski horses), and draft (Sokolski and Sztumski horses). The assumption is that Arabian horses as being under strong selection pressure for sport performance should present deeper ROH coverage than other breeds, in particular primitive breeds. Moreover, regions of the genome that were represented by very high frequency or lack of ROH in a specific population were explored to find the potential patterns of directional selection which contribute to a better understanding of genetic differences between horse breeds and types. Additionally, genomic inbreeding coefficients (F_ROH_) were calculated, allowing for an assessment of inbreeding in the studied animals. As previously, the assumption is that Arabian horses will represent higher F_ROH_ values comparing to other breeds, especially Polish Konik and Hucul. This investigation is particularly important in the case of animals with no or insufficient pedigree information.

## 2. Materials and Methods

### 2.1. Study Material, DNA Isolation and Genotyping, Filtration of Genotypic Data

The study samples comprised genomic DNA obtained from 571 randomly selected horses (a mixture of randomly chosen males and females) belonging to six different horse breeds. Selected breeds represented three different horse types: primitive horses which consisted of Polish Konik (KP) and Hucul (HC) horses; light horses in which Arabian (AR) and Malopolski horses (MLP) were chosen; and draft horses represented by Sokolski (SOK) and Sztumski (SZTUM) horses.

DNA was isolated from blood from the jugular vein, which was collected by a veterinary doctor into a vial containing EDTA K3 (10 mL of blood per sample). Horses selected for the analysis originated from different studs. In the case of Arabian horses, the samples were collected from SK Janów, SK Michałów, and SK Białka stud farms who were our project partners. Małopolski horses’ material was also sampled in the same studs and farms of individual breeders, with their consent. In the case of Hucul horses, we obtained the samples from Gładyszów and ZDIZ PIB Odrzechowa studs. Polish Konik material originated from the Popielno Research Station, IRiŻZ PAN, and the Kalitink-PTOP Research Stations. The biological material from draft horses comprised herds participating in the Sztumski and Sokolski genetic conservation programs. Moreover, each farmer participating in the program signed a cooperation agreement with the National Research Institute of Animal Production, in which they were obliged to provide data and biological material for research purposes. More details about the origin of the horses are presented in the study by Gurgul et al. [23]. All animal procedures were in accordance with EU regulations and approved by the Local Animal Care Ethics Committee No. II in Kraków (permission number 1293/2016 in accordance with EU regulations).

To purify the genomic DNA, a Sherlock AX kit (A&A Biotechnology, Gdańsk, Poland) was used. A NanoDrop2000 was utilized to determine the concentration and purity of the obtained DNA. The genotyping analysis was done according to the standard Infinium Ultra protocol, using the Neogen Equine Community BeadChip assay (Illumina, San Diego, CA, USA).

This data is presented in detail in Table 1. The presented dataset was previously utilized in our publication on the selection signatures of horses, but filtration of the SNP dataset was utilized differently [23]. Moreover, more information regarding heterozygosity observed and expected, genetic differentiation and breed-specific signatures can be found in [23] and regarding haplotype block structure in [22].

The basic filtering of the genotypic data obtained after the scanning procedure was performed jointly for all tested breeds with both Plink v1.9 and Genome Studio software v2.0 (Illumina, San Diego, CA, USA) [28]. However, the Hardy–Weinberg equilibrium tests and ROH identification were performed separately for each of the selected breeds. The initial dataset contained 65,157 SNPs. Only animals with genotypes of more than 97% (CallRate), SNPs with GenCall quality coefficients above 0.7, and GenTrain above 0.4 were used for further analysis. Subsequently, the SNPs located on the XY chromosomes and without a known genomic position (mapped to contigs) were removed. The final filtration step was a Hardy–Weinberg equilibrium test (significance threshold set to 0.0001) conducted for each breed separately. This resulted in very minor differences in the final marker panel for each breed, which was from 57,525 to 57,679 SNP markers. The average distance between markers in the dataset was around 43.1 kb (±40.1 kb).

### 2.2. Runs of Homozygosity Identification and Estimation of F_ROH_

Runs of homozygosity were identified using Plink software v1.9 [28] with the following parameters: a minimum number of 30 consecutive homozygous SNPs in ROH; a maximum distance between SNPs equal to 1 Mb; allowing 0 to 1 heterozygotes based on the ROH length; and allowing 0 to 4 missing genotypes based on ROH length. Thereafter, the identified ROH were divided into five length categories: 1–2 Mb, 2–4 Mb, 4–8 Mb, 8–16 Mb, and above 16 Mb. Based on the 0.2% assumed genotyping error for the Illumina microarrays [6] and our basic ROH calculation results for the whole dataset, we allowed 0 heterozygotes for ROH in the first four length categories (1–2 Mb, 2–4 Mb, 4–8 Mb, 8–16 Mb) and 1 heterozygote for the longest ROH length category (ROH above 16 Mb). The missing genotypes parameter was calculated according to the study of Ferencakovic et al. [13] which resulted in the following criteria per ROH length category: for the 1–2 Mb and 2–4 Mb categories, no missing SNP genotypes were permitted; for the 4–8 Mb category, 1 missing SNP was permitted; for the 8–16 Mb category, 2 missing SNPs were permitted; and for the longest ROH, (over 16 Mb) category, up to 4 missing SNP were permitted. More details regarding ROH identification are provided in an earlier study concerning cattle [5].

To calculate the differences in ROH number and the sum of ROH lengths between breeds, a Shapiro–Wilk test was used to test for normality of distribution followed by a Mann–Whitney Wilcoxon test to assess the differences. Both statistical tests were done with the use of R software version 4.1 [29].

The ROH-based measure of inbreeding (F_ROH_) was calculated according to the methodology of McQuillan et al. [2] in which the total ROH length of each individual in the selected ROH threshold category (>1 Mb, >2 Mb, >4 Mb, >8 Mb and >16 Mb) was divided by the total length of autosomal chromosomes covered by SNPs.

### 2.3. Identification of ROH Islands and Regions of No ROH Presence

Runs of homozygosity patterns were calculated based on Plink output files containing information on the number of SNPs that were present in the ROH of a given horse population. Then, 1% of the highest occurrence of SNPs were merged into regions that formed ROH islands. Regions of no ROH presence (no-ROH regions) were calculated similarly to ROH patterns, however, this time only SNPs with zero occurrences in each breed were chosen. Adjacent SNPs were merged into appropriate regions.

To check whether there is an association between ROH and recombination rate variation we have used EcuCab3 recombination map from Beesen et al. [30] for the Arabian breed and compared it with ROH patterns obtained in this study.

Lastly, overlapping genes in the respective regions were identified with the use of Ensembl BioMart software (https://www.ensembl.org/biomart/martview/a92a1775efc582c630ae5e336e20f9d3, accessed on 20 June 2022) [31], based on Ensembl genes version 101 and EquCab3.0 genome assembly. To identify molecular functions and related biological processes, the Panther Classification System (http://www.pantherdb.org/; accessed on 20 June 2022) was used. Furthermore, ROH islands and no-ROH regions were compared between breeds and between horse types.

## 3. Results

### 3.1. ROH Characteristics

**Figure 1:**
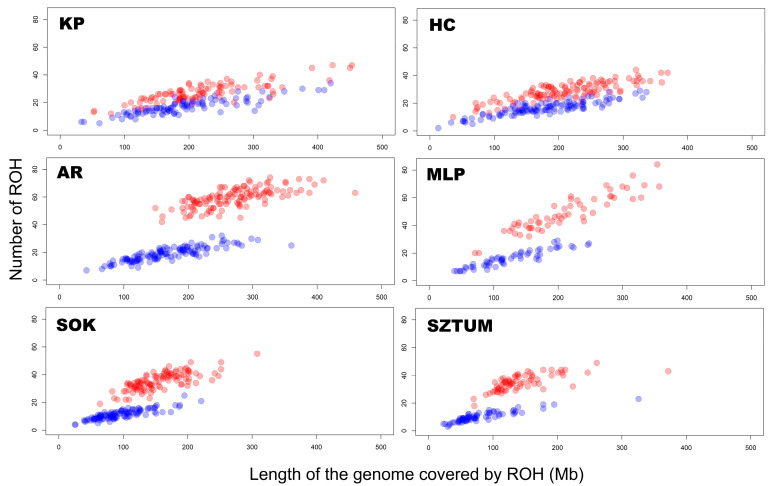
The number and length of runs of homozygosity per animal for each horse breed and type. The blue color represents all ROH, while the red color represents ROH above 4 Mb.

**Figure 2:**
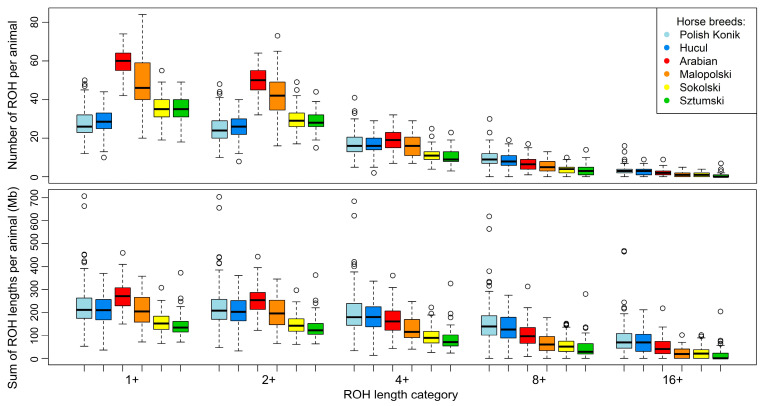
Box plot representing basic statistics of runs of homozygosity length and number in selected horse breeds.

To define ROH characteristics in an analyzed population, it is essential to focus on the number and sum of ROH lengths per animal. In the case of the analyzed horse breeds, the highest mean number of total ROH per animal was observed in light horses represented by the Arabian (59.5) and Malopolski (48.9) breeds, followed by the draft horses, Sztumski (35.0) and Sokolski (35.4). The lowest number of all ROH was observed in the case of primitive horses, namely Polish Konik (27.6) and Hucul (28.5). However, the values differ in the case of ROH calculated with the minimal length set to 4 Mb. Again, light horses presented the highest average values: 19.0 for Arabian horses and 18.0 for Malopolski horses, followed by 17.3 for the Polish Konik and 16.4 for the Hucul primitive horses. The lowest ROH values were observed for draft horses, e.g., Sokolski (11.4) and Sztumski (10.2). The basic statistics regarding these observations are graphically presented in Figure 1 and Figure 2, and in detail in Supplementary Table S1.

When the number of ROH in different breeds was analyzed, we observed significant differences (for all ROH) between most horse breeds—except for comparisons of KP vs. HC and SOK vs. SZTUM. However, in the case of ROH with lengths above 4 Mb, significant differences were observed between SOK and all breeds, as well as between SZTUM and all other breeds. In addition, comparisons of KP vs. AR, HC vs. AR, and AR vs. MLP also revealed significant differences after Mann–Whitney Wilcoxon test. These results are presented in Supplementary Table S2.

For individual animals, ROH number followed the same trend as the sum of ROH lengths. The highest mean sum of all ROH lengths was observed for Arabian horses (271.1 Mb), closely followed by KP (229.5 Mb), MLP (211.6 Mb), and HC horses (210.2 Mb). Draft horses represented the lowest average sums of all ROH lengths—157.3 Mb for Sokolski and 145.0 Mb for Sztumski horses. In the mean sum of ROH lengths data with lengths above 4 Mb, the highest values were recorded in primitive horses (KP—203.2 Mb; HC—178.8 Mb), followed by light horses (AR—168.7 Mb; MLP—127.5 Mb). The lowest average sums of ROH lengths were observed in the draft horses (SOK—96.5 Mb; SZTUM—82.7 Mb). The basic statistics for these observations are graphically presented in Figure 1 and Figure 2, and in detail in Supplementary Table S1.

In the analysis of the sum of ROH lengths per animal, we observed significant differences (for all ROH) between all horse breeds and the Sokolski and Sztumski breeds. Moreover, Arabian horses showed significant differences in comparison with both primitive breeds and with Malopolski horses. In the case of ROH above 4 Mb, significant differences were again found between all breeds and the Sokolski and Sztumski breeds, but this time also included the Malopolski horse. What is more, one of the primitive breeds, Polish Konik, was found to be statistically different from Arabian horses when considering the sum of ROH lengths above 4 Mb. These results are presented in Supplementary Table S3.

**Figure 3:**
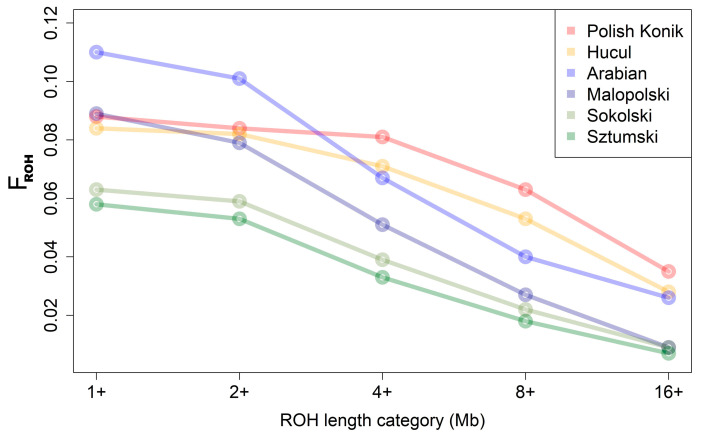
F_ROH_ for the analyzed breeds.

Mean F_ROH_ values showed the same trend in characteristics as the mean sums of ROH lengths per animal, with the highest mean values obtained for light horses: Arabian (0.110) and Malopolski (0.089). Intermediate F_ROH_ values were identified for the primitive horses, Polish Konik (0.088) and Hucul (0.084), followed by relatively lower values in the draft horses, Sokolski (0.063) and Sztumski (0.058) (Figure 3).

In the case of F_ROH_ calculated with ROH above 4 Mb, however, the results differed and the highest mean values of F_ROH_ were recorded for primitive horses (KP—0.081; HC—0.071), followed by light horses (AR—0.067; MLP—0.051). The lowest values were identified for draft horses (SOK—0.039; SZTUM—0.033). These observations are graphically presented in Figure 3 and in Supplementary Table S4.

### 3.2. Identification of ROH Islands and No-ROH Regions

**Figure 4:**
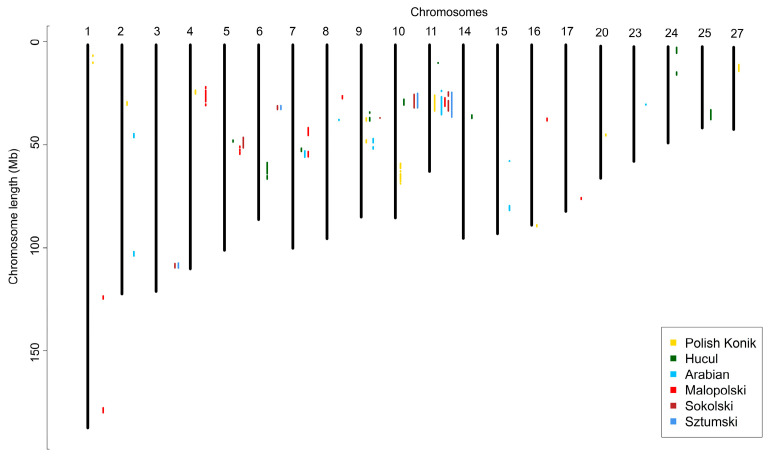
Runs of homozygosity islands detected in analyzed breeds.

Runs of homozygosity islands, representing genomic regions potentially under selection pressure, were identified for all six breeds. The most commonly occurring SNPs in ROH created from 8 to 14 ROH islands were investigated per analyzed breed. The highest number of these regions was observed in primitive (KP—14; HC—12) and light (AR—12; MLP—14) horses, while the lowest number was shown for draft horses (SOK—7; SZTUM—8). Considering the size of the presented patterns, the longest regions were those found in draft horses (SOK—from 0.3 to 6.6 Mb and from 10 to 180 SNPs; SZTUM—from 0.4 to 8.4 Mb and from 15 to 233 SNPs), followed by Polish Konik (from 0.4 to 7.7 Mb and from 8 to 198 SNPs), and Arabian horses (from 0.2 to 6.2 Mb and from 4 to 174 SNPs). The shortest regions were identified in Hucul horses (from 0.3 to 5.5 Mb and from 4 to 142 SNPs) and Malopolski horses (from 0.6 to 4.1 Mb and from 21 to 115 SNPs). The results are shown graphically in Figure 4 and in detail in Supplementary Table S5 and Figure S1.

Regions with a high occurrence of ROH overlapped with a vast number of identified genes—from 76 to 159, depending on the breed. The highest number of genes located within ROH islands were obtained for HC and SZTUM breeds—159 and 137, respectively. This was followed by SOK (121) and KP (110) horses, while lower numbers were visible for AR (90) and MLP (76) breeds. Detailed results are shown in Supplementary Table S6.

**Figure 5:**
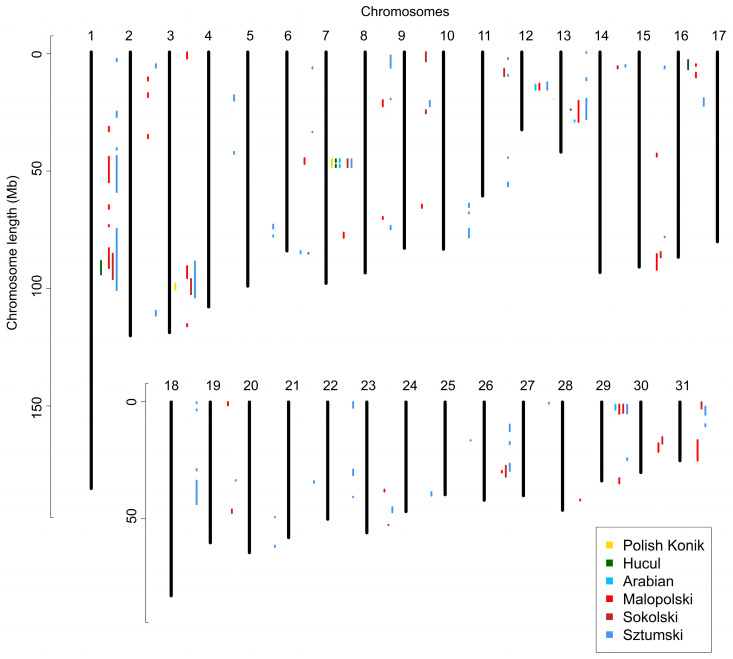
Regions of no ROH presence detected in the analyzed breeds.

Regions of no-ROH were calculated for all six breeds, by merging the adjacent SNPs with zero ROH occurrences. This led to the identification of between 2 to 57 no-ROH regions, while the number of regions was strongly linked to the number of individuals in each breed. The lowest number of regions was observed for primitive horses (KP—2; HC—5), followed by AR (6) and SOK (15) breeds. The highest number of regions was shown for SZTUM (57) and MLP (32) horses, however, both breeds represented the lowest number of individuals for which ROH was detected. When considering the size of no-ROH regions, the longest was found in draft horses (SZTUM—from 0.2 to 26.3 Mb and from 7 to 276 SNPs; SOK—from 0.1 to 10.9 Mb and from 5 to 145 SNPs), followed by MLP (from 0.5 to 8.9 Mb and from 9 to 188 SNPs) and HC horses (from 0.2 to 5.8 Mb and from 5 to 74 SNPs), and then by KP (from 2.4 to 3.3 Mb and from 16 to 29 SNPs) and AR horses (0.6 to 2.2 Mb and from 8 to 26 SNPs). The results are shown in Figure 5 and more detail is provided in Supplementary Table S7.

In the identified no-ROH regions we observed 36 to 431 genes, with the highest number of genes detected for SZTUM (431), MLP (236), SOK (148), HC (82), AR (43), and KP (36), respectively. A relatively high number of genes were common for multiple breeds. We observed 27 genes that were identified in five breeds, 9 in four breeds, and multiple variations of genes in three (from 1 to 25) and two (from 3 to 67) of the breeds. The genes for up to three breeds are presented in Table 2, with more detail in Supplementary Table S8.

**Figure 6:**
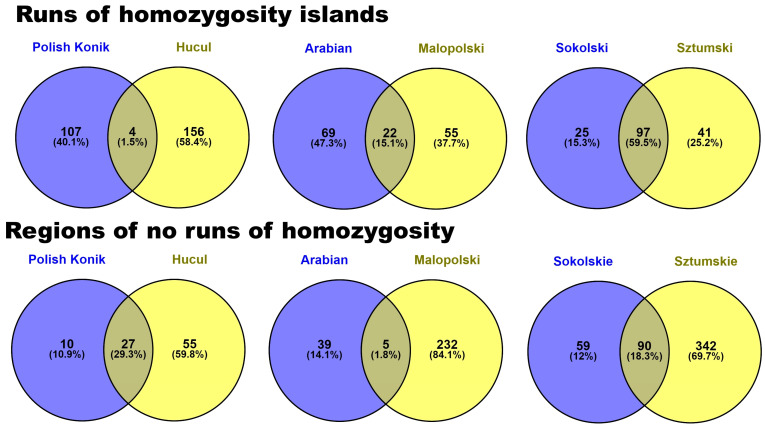
Common genes identified in ROH islands and no-ROH regions between horse breeds in regard to horse type.

Moreover, to present differences and similarities of horse breeds in regard to horse type common genes between horse breeds both for ROH islands and no-ROH regions were calculated and presented in a form of Venn diagram—Figure 6, and in more detail Supplementary Tables S6 and S8.

**Figure 7:**
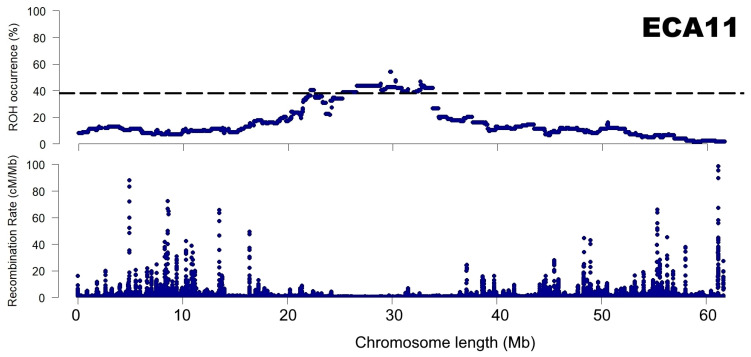
A relationship between recombination rate and ROH patterns presented for chromosome 11 for the Arabian breed.

Lastly, a comparison of EcuCab3 recombination map and ROH patterns for Arabian breed was proposed, which showed a visible relationship between ROH islands and recombination rate. This phenomena was especially evident in the case of chromosome 11 which is presented in Figure 7. All other chromosomes for which ROH islands and no-ROH regions were identified are presented in Supplementary Figure S2.

## 4. Discussion

### 4.1. ROH Characteristics

In general, the results obtained in this study for Polish horse breeds regarding ROH characteristics agree with the results of other worldwide breeds. Grilz-Seger et al. [32] identified ROH for Lipizzan horses originating from different countries based on a 700k Affymetrix microarray. The authors showed that the average sum of ROH lengths per animal for the combined dataset of Lipizzan horses was 202.1 Mb—ranging from 158.4 Mb to 211 Mb based on the country of origin. In addition, the average number of ROH per animal ranged from 171.5 to 342.9 (mean 202.1), while F_ROH_ values ranged from 0.07 to 0.15 (mean 0.13). In other research by Grilz-Seger et al. [33], using the same microarray panel, the authors presented the results of ROH identification for Slovenian Haflinger horses. Their results were as follows: average sum of ROH length per animal—270.4 Mb; average number of ROH per animal—155.6; and average F_ROH_ value—0.12. Both breeds presented by the authors belong to the light horse type and their results of the average sum of ROH lengths and F_ROH_ values are comparable to those obtained in our study. There was a vast difference in the average number of identified ROH, but this is mostly due to the microarray panel used and its density (700k Affymetrix SNP array for Grilz-Seger et al. [32,33] vs. 65k Illumina SNP array used in this study). Higher density panels allow for the identification of very short ROH which have a high influence on the average number of ROH. However, due to being very short, these do not extensively influence the average sum of ROH lengths or F_ROH_ values [1,5].

The research of Druml et al. [34], again based on a 700k Affymetrix microarray, reported results of ROH identification for various horse breeds and types. The results for the average sum of ROH lengths and ROH number, as well as F_ROH_ values of light horses, were as follows: Haflinger Austria—282. Mb, 208.5, 0.126; Haflinger Italy—316.7 Mb, 188.3, 0.141; Gidran—321.9 Mb, 217.1, 0.144; Shagya Arabian—355.1 Mb, 259, 0.158; and Purebred Arabian—396.5 Mb, 278.5, 0.177. Draft horses represented by the Noriker breed were characterized by lower values—227.5 Mb, 165, 0.101. These results are generally higher than the ones obtained in our study, likely because of the microarray panel used. However, they represent the same trend for draft horses being characterized by a lower average sum of ROH length, ROH number, and F_ROH_ values than in light horses.

Another interesting study was proposed by Gorssen at al. [35] who evaluated ROH distribution for various animal species including horse breeds. Only Arabian breed was common to breeds presented in this study, however, Gorssen et al. in their research provided SNP occurrences which allowed us to calculate ROH islands in the exact manner as presented in our study. Three of seven ROH islands calculated based on Gorssen et al. [35] overlapped with the ROH islands presented in this manuscript (ROH island located on chr 2: 99,507,282 to 103,514,544; chr 7: 44,527,348 to 52,739,455; chr 11: 26,522,101 to 30,438,602), however, it is worthy to notice that Arabian horses used in Gorssen at al. [35] represented a small population size of only 24 animals which could bias the results.

It is evident from the results obtained that, in comparison to other breeds, AR horses were characterized by a higher sum of ROH lengths per animal and higher F_ROH_ values—especially when compared with draft horses. This is probably due to the Arabian horse breeding specificity (a relatively small, closed population) and strong directional selection which reduces genetic variability and influences inbreeding values [36]. However, when carefully examining F_ROH_ values above 8 Mb, primitive breeds were characterized by even higher F_ROH_ than AR. This observation shows that KP and HC breeds were affected by recent inbreeding events that are not visible in other horse breeds [37]. This is probably because of conservative breeding, and the breeding populations experiencing population bottlenecks in the near history [38].

### 4.2. ROH Patterns

Genes found in genome regions with a high frequency of ROH are considered to be under selection pressure and thus can be good candidates for QTLs. Using the ROH island detection approach, we identified many genes which showed potential association with breeding traits. Among the genes, we distinguished WFIKNN2, CACNA1G, STXBP4, and NOG overlapping with ROH islands in four out of the six breeds. these genes are related to muscle and skeletal functions. For example, the WFIKKN2 (WAP, Follistatin/kazal, Immunoglobulin, Kunitz, and Netrin domain-containing) protein is well known for its function in muscle and skeletal tissues, namely, the inhibition of certain members of the transforming growth factor beta (TGFB) superfamily, e.g., myostatin (MSTN) and growth and differentiation factor 11 (GDF11) [39,40]. Haidet et al. [41] were the first to show an increase in muscle mass and strength in vivo, consistent with MSTN inhibition. Further, inhibition of the canonical MSTN-Smad pathway by WFIKKN2 was demonstrated in an in vitro myoblast cell line and led to an increase in myoblast differentiation and proliferation [42]. STXBP4 (syntaxin binding protein 4) binds to syntaxin (STX4) which is highly expressed in skeletal muscle and plays a critical role in insulin-stimulated glucose uptake through promoting translocation of glucose transporter 4 (GLUT4) [43]. Overexpression of STX4 enhances myogenic differentiation via the regulation of promyogenic signaling molecules Cdo and p38MAPK. Stx4 and Cdo interact physically in differentiating myoblasts. This interaction is mediated by the t-SNARE domain of Stx4, which is critical for the promyogenic function of Stx4 [44]. The noggin protein encoded by the NOG gene promotes myogenesis by inhibiting bone morphogenetic proteins (BMPs). Furthermore, noggin causes an increase in the generation of satellite cells (SCs) through Smad/Pax 7 signaling. SCs are responsible for skeletal muscle growth and repair in mammals [45,46]. Voltage-Dependent Calcium Channels (VDCCs) such as CACNA1G are involved in the influx of calcium ions into excitable cells in response to membrane depolarization. VDCCs have different roles in calcium-dependent processes, including muscle contraction, neurotransmitter release, regulation of specific genes, and gene expression [47]. Despite various genes being identified in our ROH analysis, functional validation will be necessary for the future.

In this study, we also observed numerous ROH islands in individual horse breeds which may represent ancient and ongoing breed-specific selection events. One example of such a region in Arabian horses can be a central sequence of chromosome 7 within which the Olfactomedin 2 gene (*OLFM2*) is located. This gene is responsible for controlling smooth muscle differentiation via the modification of transforming growth factor-β activity [48]. Moreover, *OLFM2* glycoprotein is highly expressed in central and peripheral nervous tissues, but it can also be involved in bone cell and muscle formation [49]. Another potentially interesting gene, uniquely observed in AR, is the *CDC37* gene. In the research of Bryan et al. [50], this was considered one of the main genes belonging to the exercise response network in horses. *CDC37* plays a key role in modifying exercise phenotypes through binding with chaperone protein Hsp90 and controlling immune regulation, cell protection, and regeneration [51]. The next identified gene, *DNMT1*, encoded DNA (cytosine-5)-methyltransferase 1 and can be strongly related to exercise adaptation. The rates of DNA methylation and epigenetic modification were also recognized as critical adjustments to effort in equine athletes [52]. What is more, exercise-induced DNA methylation changes allow for optimal adaptation to exercise [53]. We identified one more interesting ROH island unique for Arabian horses, near two long non-coding RNA transcripts (ENSECAG00000042943). The exact role of lncRNAs is still unknown, but many studies confirmed their significant function in organ development [54], epigenetic modification [55], and many others. The latest reports indicated that lncRNA and miRNAs can regulate exercise-induced adaptations via the control of physical training-related changes [56]. In humans, it has been established that selected lncRNAs were significantly up-regulated during high-intensity interval training and can modify muscle glucose metabolism, skeletal muscle differentiation, and muscle self-renewal after injury [56,57]. Considering the function of the genes described above, we can speculate that these genomic regions in Arabian horses could be related to the breed’s excellent adaptation to endurance riding and racing. Future research should investigate the identified genes in terms of possible usage as genetic markers associated with sporting results.

The next potentially interesting ROH island was detected on chromosome 10 in horses representing draft breeds (SZTUM and SOK) and Hucul horses. This region spanned a set of 12 genes belonging to the zinc finger protein family (*ZNF134*, *ZNF135*, *ZNF304*, *ZNF324*, *ZNF446*, *ZNF543*, *ZNF544*, *ZNF551*, *ZNF584*, *ZNF671*, *ZNF773*, *ZNF8*) which are known to have a broad spectrum of molecular functions. The exact role of the ZNF protein family in adaptation and selection processes is still unknown, but it has been thought to be involved in evolution [58,59]. The study of Emerson and Thomas [59] indicated that the quantity and variation among ZNF proteins rapidly increased through evolution in humans. Moreover, the authors observed the emergence of new zinc finger genes playing different functions than the genes from which they arose. Because ZNFs are one of the key factors regulating muscle growth and differentiation [60], the identified set of genes could be associated with selection pressure towards the strength, stamina, and high percentage of muscle tissue observed in the bodies of horses—especially draft breeds.

Another example of a unique ROH island observed for only draft horses (SZTUM and SOK) was identified on chromosome 3. This region included only two genes *QDPR* and *FAM184B.* Interestingly, the *FAM184B* (Family with Sequence Similarity 184 Member B) locus has been previously considered to be associated with different body construction features and locomotive traits in Spanish Purebred horses [61]. Furthermore, both genes are localized within the QTL region, which is related to conformational traits such as withers height [62]. This ROH region was identified only in SZTUM and SOK breeds, indicating that both genes should be considered as potential genetic factors that determine exterior characteristics in draft horses. Moreover, in SOK and SZTUM horses, the *LCORL* gene was identified from ROH patterns. This gene is known to be associated with body size in a variety of species. Therefore, a lesser frequency of haplotypes in the region of *LCORL* in SOK and SZTUM horses may be associated with selection pressure towards withers height [63].

#### No ROH Regions

ROH distribution is not uniform in the genome and various unique patterns do exist in separate populations. In recent years, most of the research focused on ROH regions of high frequency. However, ROH regions with low or zero ROH frequency could be equally important. Here, we detected regions of no-ROH presence and identified the spanned genes. The no-ROH regions that were detected across multiple breeds were of the most interest. The presence of such no-ROH regions in the genome is difficult to explain, however, some genetic mechanisms can be assumed. First, it might be associated with the occurrence of recombination hotspots that maximize the nucleotide variation at certain loci [64]. Second, evolutionary selection pressure for heterozygotes could be involved (balancing selection and heterozygous advantage) [65,66]. Nevertheless, none of these mechanisms can fully explain the presence of no-ROH regions, therefore other mechanisms and their combinations are likely involved. It can also be assumed that the no-ROH regions detected in this study are ROH cold spots, as described by, e.g., Wang et al. [67]. These cold spot regions were suggested to be produced through high recombination rates and are likely enriched for variants with severe adverse effects on fitness in homozygotes [9]. This observation is partially supported by our comparison of ROH patterns calculated in this study for Arabian breed and recombination map. The relationship is clearly visible, in particular in the case of chromosome 11 (Figure 7 and Supplementary Figure S2).

Within no-ROH regions, we identified 27 genes (Supplementary Tables S7 and S8) that were common in five different breeds (AR, HC, KP, SOK, SZTUM). One of these genes is *CACNA1A*, a gene associated with muscle contractility. This gene encodes a subunit of a calcium channel that regulates Ca2 + transportation, which is crucial for muscle contraction. In humans, recent research showed that the genetic variation in this gene may lead to episodic ataxia, hypokalemic periodic paralysis, malignant hyperthermia, and other diseases [68,69,70]. It is possible that the variation in this gene, which is a key gene in calcium metabolism and muscle activity, may be undesirable and could lead to muscle tissue dysfunction. However, in their research regarding selection signatures of Swedish warmblood horses, Ablondi et al. [71] pinpointed *CACNA1A* as one of the genes which were under selection for sporting performance.

Two other genes were found in no-ROH regions of the discussed five breeds—*ASF1B* and *DNAJB1*. Both these genes are associated with the encoding of histone chaperone proteins. Macario et al. [72] indicated that over 15 disorders associated with mutations in genes encoding for chaperones have been identified. Thus, variation in genes encoding for chaperone proteins may lead to many dysfunctions. In addition, selection events are known to eliminate variability, which could lead to the identification of these two genes.

Out of the 27 genes detected in regions characterized by no-ROH presence in the five horse breeds, a couple of microRNA genes were found. The *miR-23a–miR-27a–miR-24-2* comprises two paralogous gene clusters (the intronic *miR-23b–miR-27b–miR-24-1* cluster and the intergenic *miR-23a–miR-27a–miR-24-2* cluster) located at different genomic loci in mammals [73]. Various studies have indicated that members of this cluster play important roles during cardiac and skeletal muscle development, homeostasis, and disease [74,75] expression. For example, the expression levels of *mir-24*, a microRNA that promotes skeletal muscle proliferation, were found to vastly decrease in cases of skeletal muscle fibrosis [76]. A similar phenomenon was observed in the case of *mir-23*, in which the expression levels decreased during muscle atrophy [77,78]. Furthermore, Hernandez-Torres [73] established that Srf is crucial for *miR-23a–miR-27a–miR-24-2* expression, whereas other muscle-enriched transcription factors, such as Mef2c, MyoD, MyoG, and Myf6, provide regulatory signals—both at transcriptional and post-transcriptional levels. Zhou et al. [79] determined that clusters containing *miR-24 miR-23a, miR-27a,* and *miR-8992* are located on chromosome 7 in the horse genome. In this study, we have identified the presence of *eca-miR-24-1* and *eca-miR-23a* in regions of no-ROH presence in five breeds. However, their roles need to be further clarified.

What is worth mentioning is that the Equine BeadChip used in this study is a medium-density SNP microarray, which leads to limited identification of short ROH (1–4 Mb of length) [80]. Thus, the subject of regions of no-ROH should be followed by sequencing-based approaches or high-density SNP genotyping arrays.

## 5. Conclusions

In summary, within the present study, we identified runs of homozygosity distribution in the genomes of six horse breeds representing three horse types. Both basic ROH characteristics, i.e., ROH lengths and number, and genomic inbreeding coefficients (F_ROH_), were identified. In addition, ROH islands and regions of no ROH presence were carefully examined. Results obtained can shed new light on genome regions being under selection pressure in modern horse breeds, as well as unique regions for individual breeds. We believe that the results of this study could be a basis for further work on the identification of markers that determine horse breed characteristics.

## Figures and Tables

**Table 1:** Numbers and horse types of the studied breeds.

Breed	Number of Individuals	Females	Males	Horse Type
Polish Konik	99	71	28	Primitive
Hucul	116	77	39	Primitive
Arabian	124	91	33	Light
Malopolski	56	50	6	Light
Sokolski	107	86	21	Draft
Sztumski	69	69	0	Draft

**Table 2:** Genes detected in regions of no ROH presence in multiple breeds. Up to three breeds are shown.

Breeds	Number of Genes	Gene Names
AR HC KP SOK SZTUM	27	*PODNL1 KLF1 TRMT1 DNAJB1 NDUFB7 DNASE2 ADGRE3 eca-mir-8997 CACNA1A PALM3 ASF1B GADD45GIP1 FARSA eca-mir-24-1 LYL1 PTGER1 STX10 ADGRL1 DDX39A RTBDN RFX1 BRME1 C7H19orf67 PRKACA GIPC1 eca-mir-23a eca-mir-1271b*
HC MLP SOK SZTUM	9	*MEX3B EFL1 STARD5 U6 TMC3 MAT1A DYDC1 MESD SFTPA1*
MLP SOK SZTUM	5	*LRIT1 FZD8 LRIT2 CUL2 PARD3*
HC SOK SZTUM	25	*NMB KIF7 FSD2 RCCD1 AP3S2 HOMER2 MAN2A2 IQGAP1 BTBD1 IDH2 MESP1 FES SEC11A CRTC3 FURIN CIB1 HDGFL3 BLM PEX11A eca-mir-9055 UNC45A AP3B2 WDR73 BNC1 PLIN1*
KP SOK SZTUM	9	*SEL1L3 DHPS TRIR HOOK2 CCKAR PRDX2 FBXW9 WDR83OS TNPO2*
AR MLP SZTUM	1	*OR4C5*

## Data Availability

The availability of these data are restricted because de-identified genotyping data contain information about population structure and potentially allow to detect haplotypes associated with disease traits, this data can be treated as sensitive in breeding animal populations. Most of the studs and organizations which allowed to sample their horses did not permit to share genotypes publicly. For this reason, the data underlying the results presented in the study are available from Tomasz Szmatola on behalf of the National Research Institute of Animal Production or from BIOSTRATEG project manager—Jędrzej Krupiński (jedrzej.krupinski@iz.edu.pl).

## Supplementary Materials

The following supporting information can be downloaded at: https://www.mdpi.com/article/10.3390/ani12233293/s1, Supplementary Table S1: Number and sum of lengths of ROH in the analyzed breeds; Supplementary Table S2: Statistical differences regarding ROH number (for all ROH and for ROH above 4 Mb) in respect to horse breed; Supplementary Table S3: Statistical differences regarding ROH number (for all ROH and for ROH above 4 Mb) in respect to horse breed; Supplementary Table S4: F_ROH_ for the analysed breeds; Supplementary Table S5: ROH hotspots detected in the analyzed breeds; Supplementary Figure S1: Dashed line represents top 1% of ROH occurrence in analysed breed. Supplementary Table S6: Genes observed in ROH patterns and in multiple breeds; Supplementary Table S7: F_ROH_ for the analysed breeds; Supplementary Table S8: Genes in no ROH regions and common genes in no ROH regions. Supplementary Figure S2: A relationship between recombination rate and ROH patterns presented for the Arabian breed.
