# Supplementary material for: Assessment and Distribution of Runs of Homozygosity in Horse Breeds Representing Different Utility Types
Source: Animals (Basel). 2022 Nov 25;12(23):3293. doi: 10.3390/ani12233293 (PMC9736150; doi:10.3390/ani12233293)
Supplement: Supplementary file 1 [file animals-12-03293-s001.zip › Supplementary Table S1.pdf]

Supplementary Table S1. Number and sum of lengths of ROH in the analysed breeds

Breed		Stat	ROH length category (Mb)				
			1+	2+	4+	8+	16+
KP	Number/animal	**Mean**	**27.6**	**24.7**	**17.3**	**9.6**	**3.4**
		SD	7.6	7.4	6.2	4.6	2.5
		Min	12	10	5	0	0
		Max	50	48	41	30	16
	Length/animal (Mb)	**Mean**	**229.6**	**224.5**	**203.2**	**158.6**	**87.8**
		SD	103.1	103.0	100.8	95.6	76.7
		Min	52.5	47.2	33.1	0	0
		Max	707.4	703.8	684.5	618.8	468.2
HC	Number/animal	**Mean**	**28.5**	**25.7**	**16.4**	**8.5**	**2.9**
		SD	6.6	6.0	5.1	3.7	2.0
		Min	10	8	2	0	0
		Max	44	40	29	19	9
	Length/animal (Mb)	**Mean**	**210.2**	**205.1**	**178.8**	**133.1**	**70.7**
		SD	70.4	70.0	68.3	64.0	49.1
		Min	36.2	32.3	13.1	0	0
		Max	369.7	360.4	336.1	275.3	211.1
AR	Number/animal	**Mean**	**59.5**	**49.9**	**19.0**	**6.7**	**2.0**
		SD	6.9	6.8	5.2	3.1	1.6
		Min	42	32	7	1	0
		Max	74	64	32	17	9
	Length/animal (Mb)	**Mean**	**271.1**	**253.9**	**168.7**	**100.5**	**48.7**
		SD	57.1	57.4	57.4	51.3	38.9
		Min	149.3	122.1	42.9	8.4	0
		Max	460.1	443.5	360.8	313.1	218.2
MLP	Number/animal	**Mean**	**48.9**	**41.8**	**18.0**	**5.1**	**1.0**
		SD	13.2	11.6	6.1	3.1	1.0
		Min	20	16	7	0	0
		Max	84	73	29	13	5
	Length/animal (Mb)	**Mean**	**211.6**	**198.9**	**127.5**	**67.5**	**22.2**
		SD	69.3	67.0	54.1	41.2	23.4
		Min	71.1	63.8	39.8	0	0
		Max	357.9	345.2	248.1	177.4	101.8
SOK	Number/animal	**Mean**	**35.4**	**30.0**	**11.4**	**4.0**	**1**
		SD	6.4	5.8	3.5	2.1	1
		Min	19	17	4	0	0
		Max	55	49	25	10	4
	Length/animal (Mb)	**Mean**	**157.3**	**147.4**	**96.5**	**56.0**	**24.4**
		SD	41.6	41.4	38.3	34.5	25.0
		Min	64.1	60.4	25.6	0	0
		Max	308.1	297.2	221.7	152.1	102.1
SZTUM	Number/animal	**Mean**	**35.0**	**28.9**	**10.2**	**3.2**	**0.8**
		SD	5.8	5.3	3.8	2.6	1.2
		Min	18	15	3	0	0
		Max	49	44	23	14	7
	Length/animal (Mb)	**Mean**	**145.0**	**134.2**	**82.7**	**44.6**	**17.7**
		SD	47.8	47.4	47.7	42.9	31.0
		Min	70.6	62.9	23.3	0	0
		Max	372.8	362.6	326.2	280.6	204.3
