# Supplementary material for: Assessment and Distribution of Runs of Homozygosity in Horse Breeds Representing Different Utility Types
Source: Animals (Basel). 2022 Nov 25;12(23):3293. doi: 10.3390/ani12233293 (PMC9736150; doi:10.3390/ani12233293)
Supplement: Supplementary file 1 [file animals-12-03293-s001.zip › Supplementary Table S2.pdf]

Supplementary Table S2.

A. Statistical differences regarding ROH number (for all ROH) in respect to horse breed.

	KP	HC	AR	MLP	SOK	SZTUM
KP	-	W : 4937<br>P : 0.0764	W : 40<br>P <0.0001	W : 402<br>P <0.0001	W : 2124<br>P <0.0001	W : 1361<br>P <0.0001
HC		-	W : 2<br>P <0.0001	W : 403<br>P <0.0001	W : 2812<br>P <0.0001	W : 1843<br>P <0.0001
AR			-	W : 5273<br>P <0.0001	W : 13200<br>P <0.0001	W : 8538<br>P <0.0001
MLP				-	W : 5000<br>P <0.0001	W : 3284<br>P <0.0001
SOK					-	W : 3813<br>P : 0.7123
SZTUM						-

B. Statistical differences regarding ROH number for ROH above 4 Mb in respect to horse breed.

	KP	HC	AR	MLP	SOK	SZTUM
KP	-	W : 5943<br>P : 0.6577	W : 4787<br>P : 0.0047	W : 3023<br>P : 0.3489	W : 8595<br>P <0.0001	W : 5840<br>P <0.0001
HC		-	W : 5229<br>P : 0.0003	W : 3402<br>P : 0.6152	W : 9955<br>P <0.0001	W : 6758<br>P <0.0001
AR			-	W : 4439<br>P : 0.0028	W : 11798<br>P <0.0001	W : 7847<br>P <0.0001
MLP				-	W : 4405<br>P <0.0001	W : 3061<br>P <0.0001
SOK					-	W : 4484<br>P : 0.0158
SZTUM						-

W- linear rank sum

P- pvalue of the test
