# Supplementary material for: Assessment and Distribution of Runs of Homozygosity in Horse Breeds Representing Different Utility Types
Source: Animals (Basel). 2022 Nov 25;12(23):3293. doi: 10.3390/ani12233293 (PMC9736150; doi:10.3390/ani12233293)
Supplement: Supplementary file 1 [file animals-12-03293-s001.zip › Supplementary Table S3.pdf]

Supplementary Material Table S3.

A. Statistical differences regarding sum of ROH lengths calculated for all ROH in respect to horse breed.

	KP	HC	AR	MLP	SOK	SZTUM
KP	-	W : 6057<br>P : 0.4898	W : 3549<br>P <0.0001	W : 2962<br>P : 0.4814	W : 8175<br>P <0.0001	W : 5602<br>P <0.0001
HC		-	W : 3651<br>P <0.0001	W : 3299<br>P : 0.8693	W : 9241<br>P <0.0001	W : 6329<br>P <0.0001
AR			-	W : 5161<br>P <0.0001	W : 12639<br>P <0.0001	W : 8180<br>P <0.0001
MLP				-	W : 4463<br>P <0.0001	W : 3116<br>P <0.0001
SOK					-	W : 4536<br>P : 0.0102
SZTUM						-

B. Statistical differences regarding sum of ROH lengths calculated for ROH above 4 Mb in respect to horse breed.

	KP	HC	AR	MLP	SOK	SZTUM
KP	-	W : 6286<br>P : 0.2324	W : 7348<br>P : 0.0113	W : 4236<br>P <0.0001	W : 9441<br>P <0.0001	W : 6240<br>P <0.0001
HC		-	W : 7954<br>P : 0.1567	W : 4673<br>P <0.0001	W : 10551<br>P <0.0001	W : 7057<br>P <0.0001
AR			-	W : 4847<br>P <0.0001	W : 11389<br>P <0.0001	W : 7698<br>P <0.0001
MLP				-	W : 4033<br>P : 0.0002	W : 2911<br>P <0.0001
SOK					-	W : 4747<br>P : 0.0012
SZTUM						-

W- linear rank sum

P- pvalue of the test
