# Supplementary material for: Assessment and Distribution of Runs of Homozygosity in Horse Breeds Representing Different Utility Types
Source: Animals (Basel). 2022 Nov 25;12(23):3293. doi: 10.3390/ani12233293 (PMC9736150; doi:10.3390/ani12233293)
Supplement: Supplementary file 1 [file animals-12-03293-s001.zip › Supplementary Table S4.pdf]

Supplementary Material Table S4. F_{ROH} for the analysed breeds

**Breed**	**F_{ROH}**	**ROH length category (Mb)**				
		**1+**	**2+**	**4+**	**8+**	**16+**
KP	**Mean**	**0.088**	**0.084**	**0.081**	**0.063**	**0.035**
	SD	0.041	0.041	0.040	0.038	0.031
	Min	0.021	0.019	0.013	0.000	0.000
	Max	0.282	0.281	0.273	0.247	0.187
HC	**Mean**	**0.084**	**0.082**	**0.071**	**0.053**	**0.028**
	SD	0.028	0.028	0.027	0.026	0.020
	Min	0.014	0.013	0.005	0.000	0.000
	Max	0.148	0.144	0.134	0.110	0.084
AR	**Mean**	**0.110**	**0.101**	**0.067**	**0.040**	**0.026**
	SD	0.023	0.023	0.023	0.020	0.019
	Min	0.060	0.049	0.017	0.003	0.008
	Max	0.184	0.177	0.144	0.125	0.047
MLP	**Mean**	**0.089**	**0.079**	**0.051**	**0.027**	**0.009**
	SD	0.028	0.027	0.022	0.046	0.009
	Min	0.028	0.025	0.016	0.000	0.000
	Max	0.143	0.138	0.099	0.071	0.041
SOK	**Mean**	**0.063**	**0.059**	**0.039**	**0.022**	**0.009**
	SD	0.017	0.017	0.015	0.014	0.010
	Min	0.026	0.024	0.010	0.000	0.000
	Max	0.123	0.119	0.089	0.061	0.041
SZTUM	**Mean**	**0.058**	**0.053**	**0.033**	**0.018**	**0.007**
	SD	0.019	0.019	0.019	0.017	0.012
	Min	0.028	0.025	0.009	0.000	0.000
	Max	0.149	0.145	0.130	0.112	0.082
