# Supplementary material for: Assessment and Distribution of Runs of Homozygosity in Horse Breeds Representing Different Utility Types
Source: Animals (Basel). 2022 Nov 25;12(23):3293. doi: 10.3390/ani12233293 (PMC9736150; doi:10.3390/ani12233293)
Supplement: Supplementary file 1 [file animals-12-03293-s001.zip › Supplementary Table S5.pdf]

Supplementary Table S5. ROH hotspots detected in the analyzed breeds

Breed	CHR	Start	Stop	Length (Mb)	Number of SNPs
KP	1	5,040,886	5,445,204	0.404	17
	1	8,315,402	8,967,968	0.653	20
	2	27,572,525	29,081,977	1.509	41
	4	21,973,202	23,700,482	1.727	46
	9	35,306,553	36,838,154	1.532	48
	9	45,958,978	47,434,669	1.476	42
	10	57,525,808	59,874,862	2.349	47
	10	61,112,691	61,405,011	0.292	8
	10	62,620,151	66,248,521	3.628	91
	10	67,110,137	67,451,792	0.342	10
	11	24,279,941	32,013,448	7.734	198
	16	87,269,741	88,069,785	0.800	25
	20	42,762,110	43,535,049	0.773	19
	27	8,512,990	11,588,744	3.0758	82
HC	5	46,179,705	47,148,310	0.969	29
	6	57,096,231	62,575,843	5.479	142
	6	63,532,034	65,049,128	1.517	43
	7	50,112,758	51,710,979	1.598	36
	9	32,537,122	33,058,456	0.521	10
	9	35,172,519	36,838,154	1.666	41
	10	26,437,111	29,067,063	2.629	60
	11	8,647,652	8,925,639	0.278	7
	14	33,986,115	35,708,852	1.723	49
	24	370,034	3,269,944	2.899	68
	24	12,311,336	13,795,884	1.485	7
	25	30,710,237	35,441,033	4.731	113
AR	2	43,053,164	44,813,488	1.760	53
	2	100,357,472	102,320,578	1.963	116
	7	51,342,600	54,419,009	3.076	65
	8	36,282,328	36,595,207	0.313	10
	9	45,385,652	47,396,450	2.011	54

	9	49,471,705	50,315,411	0.844	23
	11	22,131,902	22,472,610	0.341	10
	11	25,233,252	31,434,860	6.202	175
	11	31,956,077	33,792,266	1.836	61
	15	56,152,443	56,420,784	0.268	7
	15	77,908,479	80,249,933	2.341	57
	23	27,990,315	28,153,628	0.163	4
**MLP**	1	121,839,949	123,229,881	1.389	29
	1	176,071,336	178,331,664	2.260	57
	4	20,349,108	21,066,804	0.718	21
	4	21,973,373	27,521,898	5.549	133
	4	28,688,509	29,351,095	0.663	28
	5	49,104,624	49,713,098	0.608	20
	5	50,570,292	51,571,722	1.001	26
	5	52,130,295	53,025,154	0.895	23
	7	40,039,177	43,905,056	3.866	86
	7	51,770,649	54,253,799	2.483	52
	8	24,633,706	26,072,376	1.439	41
	11	25,724,472	29,829,190	4.105	115
	16	35,601,403	36,816,206	1.215	36
	17	74165930	74,910,629	0.745	21
**SOK**	3	106,138,107	108,098,923	1.961	51
	5	44,816,748	49,922,915	5.106	116
	6	29,454,070	31,320,967	1.867	48
	9	35,264,625	35,551,277	0.287	10
	10	23,934,045	30,578,626	6.645	180
	11	22,842,852	24,867,196	2.024	55
	11	27,019,831	32,013,448	4.994	135
**SZTUM**	3	105,890,193	108,201,068	2.311	57
	6	29,387,236	31,305,190	1.918	48
	10	23,514,304	30,578,626	7.064	190
	11	22,986,900	31,434,860	8.448	233
	11	31,956,077	32,309,605	0.354	18

	11	32,739,985	33,253,535	0.513	36
	11	33,670,635	34,049,633	0.379	28
	11	34,523,642	35,078,510	0.555	15
