# Supplementary material for: Assessment and Distribution of Runs of Homozygosity in Horse Breeds Representing Different Utility Types
Source: Animals (Basel). 2022 Nov 25;12(23):3293. doi: 10.3390/ani12233293 (PMC9736150; doi:10.3390/ani12233293)
Supplement: Supplementary file 1 [file animals-12-03293-s001.zip › Supplementary Table S7.pdf]

Supplement Table S7. Regions of no ROH detected in the analyzed breeds

Breed	CHR	Start	Stop	Length (Mb)	Number of SNPs
KP	3	98676818	101079941	2.403	29
	7	45673943	49023352	3.349	16
HC	1	88951573	94760030	5.808	74
	7	45673943	46860621	1.187	7
	7	47962591	49023352	1.061	9
	13	24451306	24607012	0.156	5
	16	3391246	7325274	3.934	72
AR	6	84728043	85871501	1.143	21
	7	45565125	46860621	1.295	8
	7	47962591	49023352	1.061	9
	12	13845914	16046982	2.201	26
	13	29052395	29606166	0.554	14
	29	1110269	3288100	2.178	25
MLP	1	31815839	33792526	1.976	54
	1	44679301	46543554	1.864	32
	1	47053485	55484817	8.431	149
	1	65217852	66862525	1.644	49
	1	73692254	74271323	0.579	9
	1	83519792	92098236	8.578	119
	2	10649226	12224996	1.575	37
	2	17471853	19231826	1.759	48
	2	35221301	36737662	1.516	41
	3	116670	2810638	2.693	66
	3	91209656	96398334	5.188	69
	3	115832872	116850288	1.017	30
	6	45213355	47653561	2.440	61
	7	76937713	79117880	2.180	58
	8	20460668	23126728	2.666	70
	8	70257029	71192972	0.935	29
	9	64992466	66395281	1.402	35
	12	13419192	16046982	2.627	37
	13	20774408	29752054	8.977	179
	14	6064869	6988997	0.924	26
	15	43188539	44510126	1.321	34
	15	86041135	92835963	6.794	120
	16	5203564	5970026	0.766	13
	16	8751024	10834342	2.083	34
	19	29605	1520206	1.490	32
	23	37336369	38128750	0.792	21
	26	29339592	30036230	0.696	22
	28	41496813	41977080	0.480	14
	29	1110269	5027361	3.917	61
	29	32427227	34735896	2.308	66
	30	17584029	21340121	3.756	98
	31	16241953	24906381	8.664	188
SOK	1	85849355	96749241	10.899	145
	3	96709111	99223755	2.514	37
	3	99745444	103191969	3.446	53
	6	85631025	85871501	0.240	6

	7	45673943	49023352	3.349	16
	9	53510	4029650	3.976	94
	9	24731688	26134397	1.402	40
	11	7154417	10249710	3.095	83
	15	85145230	87440663	2.295	30
	19	45792372	47310479	1.518	49
	23	52379251	52477336	0.098085	5
	26	27219932	31862561	4.642629	92
	29	1110269	4683840	3.573571	48
	30	14971894	17823866	2.851972	78
	31	102326	2823694	2.721368	77
SZTUM	1	2734928	3949461	1.214533	31
	1	25333814	27703122	2.369308	74
	1	40938716	41656526	0.71781	20
	1	44300989	59629393	15.3284	276
	1	75180142	101430570	26.25043	241
	2	5172151	6682795	1.510644	35
	2	110182872	112345392	2.16252	59
	3	89113594	104576584	15.46299	209
	4	18385118	20745865	2.360747	59
	4	42463698	43399927	0.936229	17
	5	73509948	75203575	1.693627	37
	5	78062436	78691892	0.629456	13
	6	6473778	7000935	0.527157	21
	6	33966863	34146428	0.179565	7
	7	45673943	49023352	3.349409	16
	8	1397315	3811325	2.41401	39
	8	4421459	6689480	2.268021	46
	8	19845807	20158266	0.312459	7
	8	74008845	75577532	1.568687	40
	9	20747019	23119512	2.372493	61
	10	64571101	66054929	1.483828	29
	10	68252111	68674224	0.422113	11
	10	75152109	79022148	3.870039	88
	11	2518996	3073081	0.554085	17
	11	9628365	10077744	0.449379	11
	11	44820354	45130619	0.310265	13
	11	55512127	57246218	1.734091	46
	12	12907785	16046982	3.139197	44
	13	32110	408167	0.376057	7
	13	11070524	12070381	0.999857	34
	13	19886658	28655294	8.768636	179
	14	5550688	6209813	0.659125	20
	15	6115524	6971084	0.85556	11
	15	78573644	79004432	0.430788	11
	16	19702312	22880960	3.178648	29
	18	71416	600679	0.529263	16
	18	3138457	3605878	0.467421	10
	18	28568011	29232491	0.66448	19
	18	33557443	43596515	9.96093	41
	19	33271596	33596515	0.324919	11
	20	48938506	49186160	0.247654	12
	20	61196155	61801258	0.605103	18

	21	33750764	34514375	0.763611	21
	22	9814	2544840	2.535026	72
	22	28787161	31262159	2.474998	70
	22	40381355	40697361	0.316006	7
	23	44762900	47082200	2.3193	66
	24	38330520	39955402	1.624882	41
	25	16233243	16496247	0.263004	10
	26	9593864	12548030	2.954166	51
	26	16991244	18028008	1.036764	23
	26	26271020	29435717	3.164697	54
	27	320414	820529	0.500115	7
	29	1110269	4971981	3.861712	57
	29	23912377	24817095	0.904718	20
	31	1956892	5443516	3.486624	90
	31	9466964	10424808	0.957844	24
