# Supplementary figures and images for: Assessment and Distribution of Runs of Homozygosity in Horse Breeds Representing Different Utility Types
Source: Animals (Basel). 2022 Nov 25;12(23):3293. doi: 10.3390/ani12233293 (PMC9736150; doi:10.3390/ani12233293)

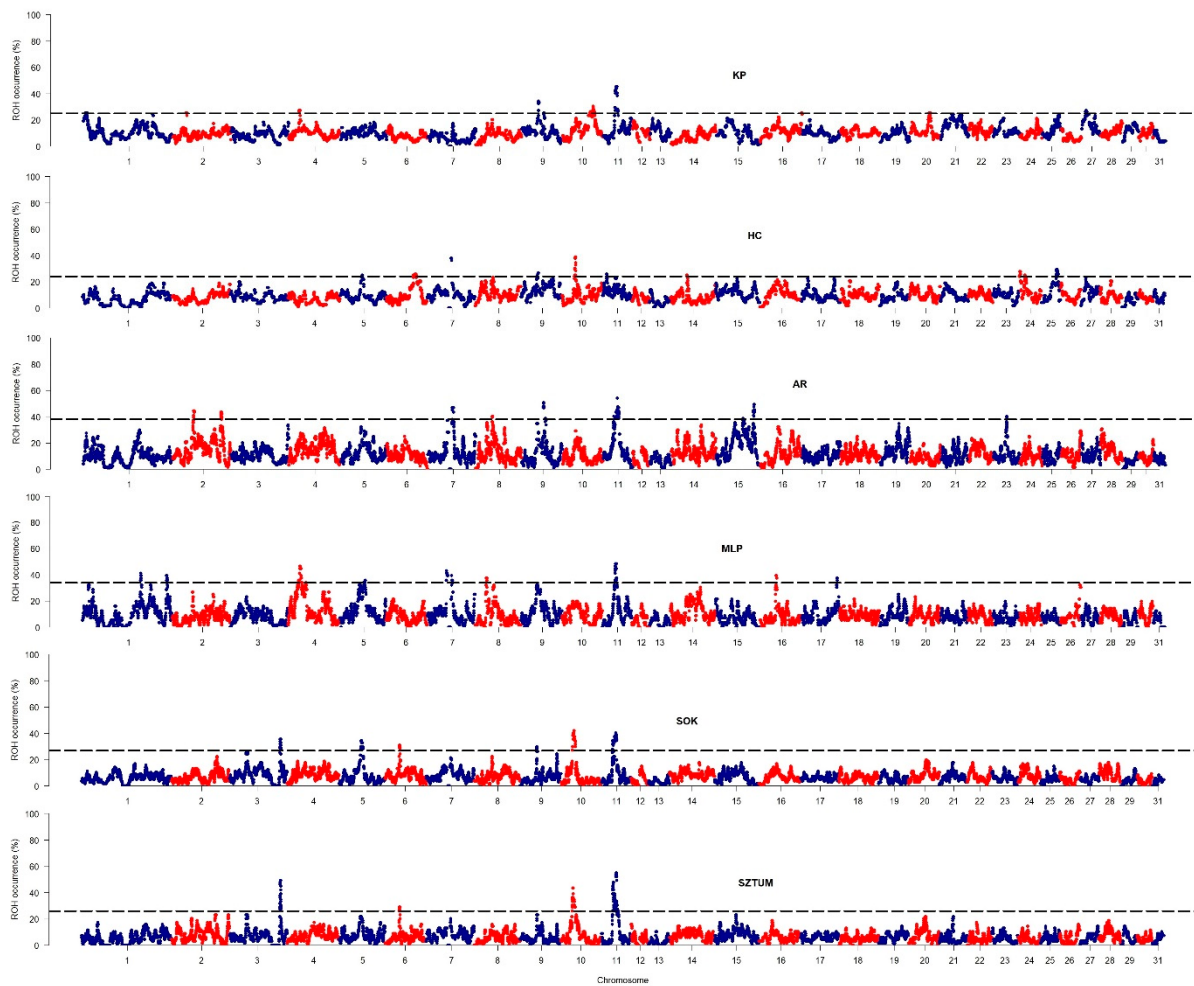

Dashed line represents top 1% of ROH occurrence in analysed breed.

Supplement: Supplementary file 1 [file animals-12-03293-s001.zip › Supplementary Figure S1.pdf]

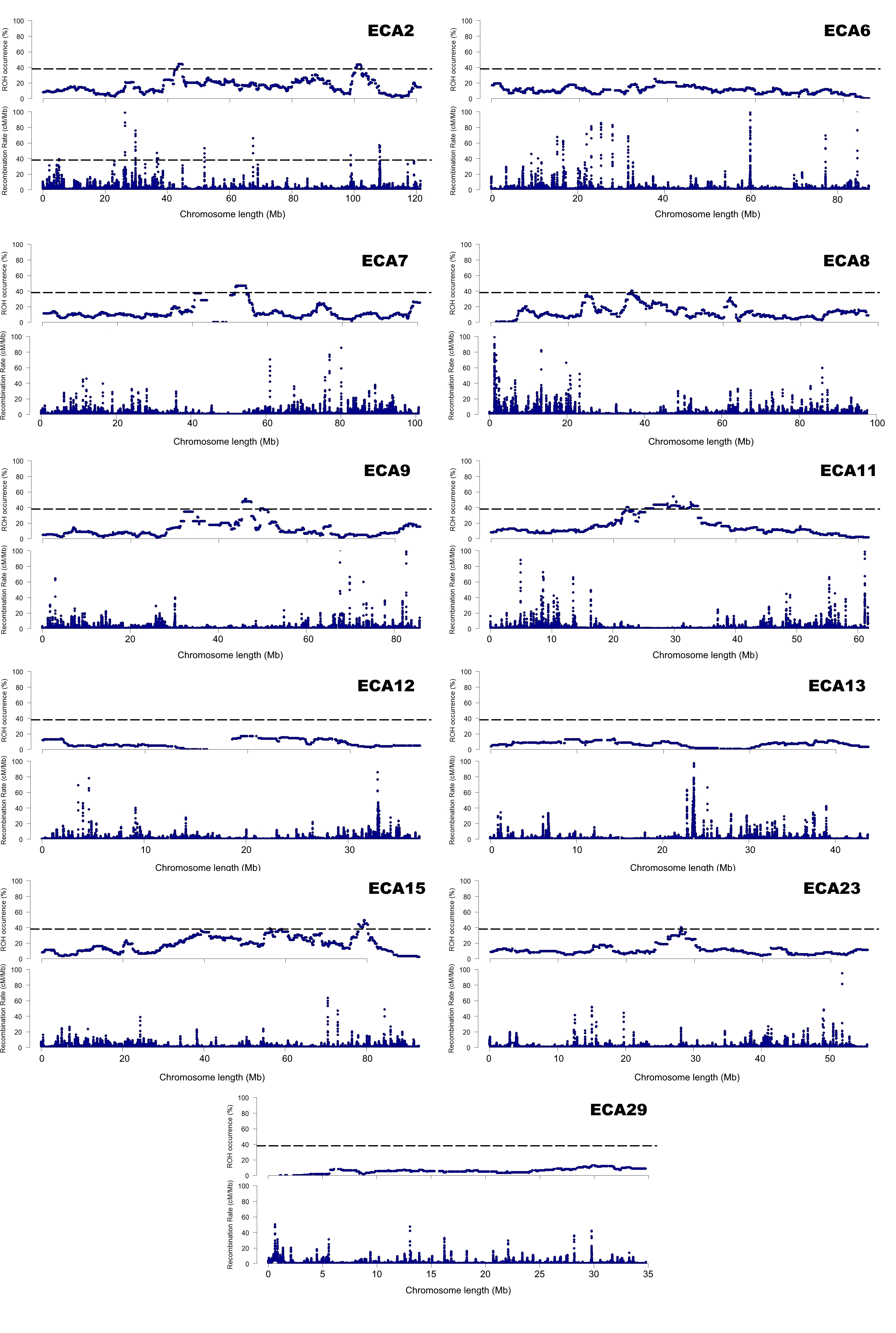

Supplement: Supplementary file 1 [file animals-12-03293-s001.zip › Supplementary Figure S2.jpg]
